# Transforming Relational Care Values in AI-Mediated Healthcare: A Text Mining Analysis of Patient Narrative

**DOI:** 10.3390/healthcare14030371

**Published:** 2026-02-02

**Authors:** So Young Lee

**Affiliations:** Department of Nursing, Kyungdong University, Wonju-si 24695, Gangwon-do, Republic of Korea; lsy@kduniv.ac.kr

**Keywords:** AI-based care, patient narratives, text mining, topic modeling, sentiment analysis, patient-centered care

## Abstract

**Background**: This study examined how patients and caregivers perceive and experience AI-based care technologies through text mining analysis. The goal was to identify major themes, sentiments, and value-oriented interpretations embedded in their narratives and to understand how these perceptions align with key dimensions of patient-centered care. **Methods**: A corpus of publicly available narratives describing experiences with AI-based care was compiled from online communities. Natural language processing techniques were applied, including descriptive term analysis, topic modeling using Latent Dirichlet Allocation, and sentiment profiling based on a Korean lexicon. Emergent topics and emotional patterns were mapped onto domains of patient-centered care such as information quality, emotional support, autonomy, and continuity. **Results**: The analysis revealed a three-phase evolution of care values over time. In the early phase of AI-mediated care, patient narratives emphasized disruption of relational care, with negative themes such as reduced human connection, privacy concerns, safety uncertainties, and usability challenges, accompanied by emotions of fear and frustration. During the transitional phase, positive themes including convenience, improved access, and reassurance from diagnostic accuracy emerged alongside persistent emotional ambivalence, reflecting uncertainty regarding responsibility and control. In the final phase, care values were restored and strengthened, with sentiment patterns shifting toward trust and relief as AI functions became supportive of clinical care, while concerns related to depersonalization and surveillance diminished. **Conclusions**: Patients and caregivers experience AI-based care as both beneficial and unsettling. Perceptions improve when AI enhances efficiency and information flow without compromising relational aspects of care. Ensuring transparency, explainability, opportunities for human contact, and strong data protections is essential for aligning AI with principles of patient-centered care. Based on a small-scale qualitative dataset of patient narratives, this study offers an exploratory, value-oriented interpretation of how relational care evolves in AI-mediated healthcare contexts. In this study, care-ethics values are used as an analytical lens to operationalize key principles of patient-centered care within AI-mediated healthcare contexts.

## 1. Introduction

Digital health technologies and artificial intelligence (AI) have become essential components of contemporary healthcare systems. Remote patient monitoring tools, AI-driven diagnostic algorithms, virtual health assistants, predictive analytics platforms, and mobile health applications promise to enhance clinical efficiency, expand access, and support more accurate data-driven decision-making. The global digital health industry continues to grow rapidly, and AI is being increasingly adopted across disease screening, treatment recommendation, patient monitoring, and chronic disease management [1]. Despite these advances, a significant gap remains in understanding how patients experience and interpret AI-based digital healthcare in meaningful ways. Existing research on medical AI has primarily focused on technological performance—clinical validity, algorithmic accuracy, implementation challenges, and clinician adoption. While these domains are crucial, they often overlook the subjective and relational dimensions of patient experience [2,3]. Patient perceptions are particularly important because expectations about trust, usefulness, transparency, and alignment with personal values strongly influence the acceptance and sustained use of digital health technologies [4]. When patients perceive AI as impersonal, opaque, or disconnected from their needs and expectations, disengagement frequently follows, undermining the intended benefits of digital health innovation [5,6]. Care has traditionally been understood as a relational practice grounded in presence, empathy, responsiveness, and the recognition of individual dignity and vulnerability [7]. As AI systems increasingly mediate, augment, or even replace aspects of patient–provider interaction, fundamental questions arise about how such shifts reshape the meaning and experience of care. In particular, it remains unclear whether AI enhances the quality and reach of care or diminishes its human connection. Patient narratives generated in online health communities, social media platforms, digital health forums, and peer-support spaces offer valuable insight into how individuals perceive and experience AI-mediated care in real-world contexts. Text mining methodologies enable systematic analysis of such large-scale narrative datasets while preserving the richness of individual patient voices [3]. Diagnostic comprehensive text mining analysis of patient-generated narratives to explore perceptions and experiences of AI-based digital healthcare technologies. Specifically, it examines how patients describe and interpret their interactions with AI-enabled tools, the values and concerns that emerge in their narratives, and how emotional expressions reflect broader understandings of care, trust, autonomy, and human connection.

In the Korean healthcare context, these questions hold particular significance. South Korea is among the most rapidly digitalizing medical environments, characterized by high patient volumes, widespread adoption of AI-assisted diagnostic tools, and strong societal expectations for efficiency and accuracy [1]. During the COVID-19 pandemic, telemedicine was temporarily legalized and later restricted, creating a distinctive landscape in which patients and clinicians navigated rapidly shifting modes of digital interaction [5]. This context underscores the need for an in-depth, patient-centered investigation of AI-enabled care experiences in Korea. To clarify the novelty of the present study, Table 1 compares its focus, data sources, methodological approach, and key findings with recent studies on patient perceptions of AI in healthcare (Table 1).

## 2. Materials and Methods

### 2.1. Study Design

This study employed a text mining approach to analyze patient narratives related to AI-mediated care. Automated topic modeling was combined with manual thematic review to capture key interpretive patterns in how patients understood AI-enabled healthcare. The analysis was guided by the five core values of care ethics—attentiveness, responsibility, competence, responsiveness, and trust—to trace shifts in the meaning of care over time.

### 2.2. Data Sources and Data Collection

Patient narratives were obtained from publicly accessible online platforms, including health forums, patient review sites, and healthcare-related social media communities in which individuals discussed their experiences with AI-based medical technologies. A total of 25 narrative cases were collected from three sources—public news comments, healthcare provider forums, and patient communities—each contributing unique perspectives on public perceptions, clinical experiences, and patient concerns (Table 2).

Data were collected from March 2020 to August 2025 and categorized into three phases reflecting the progression of digital healthcare transformation.

Phase 1 (2020–2021): Early COVID-19 and the abrupt expansion of remote care.

Phase 2 (2022–2023): Transition period characterized by normalization of remote care and growing clinical adoption of AI.

Phase 3 (2024–2025): Stabilization and integration of AI technologies into healthcare workflows.

From a larger pool of publicly available online posts initially screened across the three platforms, narratives were purposefully selected to ensure analytic depth rather than representativeness. The final sample of 25 narratives was determined through an iterative review process based on the clarity of experiential description, explicit reference to AI-mediated care technologies, and relevance to relational care values. Posts that were promotional, fragmented, repetitive, or lacked first-hand experiential content were excluded. This purposive sampling strategy is consistent with qualitative and interpretive text mining approaches that prioritize conceptual richness and longitudinal interpretability over sample size.

Narratives were included if they described direct experiences with AI or digital healthcare technologies, were written in Korean, and were publicly accessible. Posts containing advertisements, unclear or fragmented content, or personally identifiable information were excluded.

### 2.3. Data Preprocessing and Analytical Procedures

All textual data were preprocessed by removing URLs, special characters, noninformative elements, and duplicate entries, after which the cleaned narratives were standardized and segmented into analyzable units using Python-based tools such as NLTK (version 3.8.1) and spaCy (version 3.7). The analytical procedures combined topic modeling, thematic coding, sentiment analysis, and comparative analysis across subjects and phases.

Narratives were first coded according to the five care ethics values—attentiveness, responsibility, competence, responsiveness, and trust—to examine how each value was preserved, weakened, or reinterpreted within AI-mediated healthcare contexts. Shifts in these values were then classified into six categories: weakened, strengthened, transformed, emerging, ambiguous, and unresolved, allowing systematic identification of the direction and nature of change.

Topic modeling using Latent Dirichlet Allocation (LDA) was conducted to extract major thematic domains. These automatically generated topics were refined through iterative thematic review and organized into clusters reflecting perceptions of care, trust, autonomy, and human–technology interaction. Sentiment analysis, based on a Korean sentiment lexicon, was applied to classify emotional polarity (positive, negative, neutral) and assess the emotional tone associated with AI-mediated care.

Finally, subject-specific and temporal analyses were performed. Patient and provider narratives were examined separately to distinguish differences in perspectives, and the three phases were compared to trace the evolving trajectory of care values throughout the broader digital transition in healthcare. To enhance the credibility of manual coding, an iterative coding process was employed. Initial coding based on care ethics values was followed by repeated re-examination of narratives across phases to ensure internal consistency and conceptual coherence. Coding decisions were continuously compared across cases and time periods, and discrepancies or ambiguities were resolved through reflexive review rather than automated assignment. This process functioned as an internal validation strategy in the absence of formal inter-rater agreement.

### 2.4. Quantitative Metrics, Data Availability, and Ethics

Quantitative analysis included word-frequency distributions, term co-occurrence networks, thematic prevalence, and sentiment patterns generated using Python-based text mining tools (Python 3.10). All data were publicly accessible, fully de-identified, and compliant with relevant research ethics guidelines, allowing informed consent to be waived. Analysis code will be made available on GitHub, while raw narrative data cannot be released due to platform usage policies. However, aggregated thematic results and complete methodological documentation are provided to ensure transparency and reproducibility.

### 2.5. Use of Generative Artificial Intelligence

Generative AI tools, specifically ChatGPT (OpenAI, GPT-4), were used to enhance academic clarity, summarize relevant literature, and support the initial development of coding frameworks and analytical structures. All AI-assisted outputs were critically reviewed and validated by the researchers, who maintained full responsibility for all analytical interpretations and conclusions. To ensure methodological rigor, the use of generative AI was strictly limited to supportive functions, including literature summarization, language refinement, and the preliminary organization of coding frameworks. All interpretive, analytical, and ethical judgments—such as the identification of care values, the classification of value shifts, and the interpretation of thematic and sentiment patterns—were conducted exclusively by the author. Generative AI outputs were treated as provisional aids rather than analytical evidence and were critically reviewed, revised, or discarded as necessary. At no stage did AI systems participate in interpretive decision-making, data labeling, or theoretical inference.

## 3. Results

### 3.1. Overall Patterns of Change in Care Values

Analysis of the 25 narrative cases revealed six types of change in care values, with weakened and strengthened patterns appearing most frequently, alongside ambiguous, transformed, emerging, and unresolved forms of change. In Phase 1, care values were predominantly weakened, reflecting the disruption caused by the sudden shift to non-contact care. During Phase 2, a more diverse pattern of change emerged, including redefinition, ambiguity, and the appearance of new forms of care. By Phase 3, all cases demonstrated strengthened care values, indicating a restorative trajectory in which values diminished during the crisis gradually recovered and were reinforced as AI technologies became stabilized and integrated into clinical practice.

### 3.2. Phase-Specific Characteristics

The analysis revealed distinct shifts in care values across the three phases, reflecting how the meaning and practice of care evolved through crisis, adaptation, and eventual integration with AI technologies. Table 3 summarizes these phase-specific characteristics by organizing the dominant change patterns, key value shifts, and representative quotations from patients and healthcare providers. This structured comparison highlights how each phase embodies a unique configuration of relational, ethical, and technical challenges in the delivery of care (Table 3).

### 3.3. Reinterpretation of Care Values Across

Analysis of the five care ethics values across the three phases revealed distinct patterns of transition and reinterpretation. These shifts are summarized in Table 4.

### 3.4. Differences Between Patients and Providers

Analysis of stakeholder-specific perspectives revealed clear differences between patients and healthcare providers in how they interpreted the impact of AI on care.

Among patients (*n* = 17), attentiveness, responsibility, and trust emerged as the most salient care values. Their concerns centered on whether clinicians were truly “seeing” them in a mediated environment, who would be accountable when AI was involved in clinical decisions, and whether AI or human clinicians should be trusted more in moments of uncertainty. These concerns highlight that, for patients, care is fundamentally anchored in stability, visibility, and trustworthiness.

In contrast, providers (*n* = 8) emphasized attentiveness, responsibility, and competence as their primary care values. Their reflections focused on navigating shifting professional roles, redefining the competencies required in an AI-integrated clinical environment, and exploring the possibility of sustaining relational care despite technological mediation. For providers, the introduction of AI brought forth challenges related to role identity, professional expertise, and the maintenance of human-centered care practices.

Overall, patients tended to prioritize the security and reliability of care, whereas providers were more concerned with role adaptation and professional transformation in the evolving technological landscape.

## 4. Discussion

The quantitative patterns observed are interpreted as descriptive tendencies within a small qualitative corpus, with primary emphasis placed on thematic and value-based interpretation. The findings of this study indicate that the integration of artificial intelligence and digital health technologies into clinical practice produces a dynamic reconfiguration of care values over time. Rather than simply enhancing or diminishing specific aspects of care, AI-mediated environments reshaped the meaning, expression, and relational foundations of care itself. The three-phase trajectory—from disruption in the early pandemic period, to uncertainty during the transition to AI-enabled workflows, to reintegration as digital systems stabilized—reflects how the experience of care evolves within rapidly transforming technological settings.

In the initial phase, the sudden expansion of remote care resulted in a widespread weakening of traditional care values. Patients frequently reported feeling unseen or insufficiently examined, while providers experienced reduced opportunities for relational engagement. These perceptions align with prior evidence that early telemedicine environments limit clinicians’ ability to convey empathy, assess subtle cues, or establish trust. In this period, attentiveness, trust, and responsibility were disrupted not because clinicians lacked commitment, but because the medium of care constrained the relational practices traditionally associated with therapeutic encounters [8,9].

As AI technologies became more visible within clinical workflows, narratives from Phase 2 reflected a complex landscape of uncertainty, redefinition, and emerging forms of care. Patients expressed significant concern regarding accountability, particularly when AI tools contributed to diagnostic decisions. Providers, meanwhile, described shifts in their professional roles as they increasingly interpreted AI outputs rather than functioning solely as diagnosticians. These findings echo global debates about liability, transparency, and the redistribution of clinical authority in AI-assisted healthcare [10,11]. Importantly, this period marked the beginning of new hybrid practices in which clinicians combined technological insights with relational judgment, although the coherence of these practices was not yet fully established.

By Phase 3, all care values exhibited strengthened patterns, suggesting a restorative and integrative trajectory. As AI tools became more reliable and institutional structures clarified lines of responsibility, both patients and providers experienced greater stability and confidence in AI-enabled care. Clinicians described having more time to focus on patients’ emotions and life contexts, while patients reported enhanced reassurance when AI accuracy was combined with personalized explanation. These findings suggest that, when appropriately integrated, AI can alleviate cognitive and administrative burdens, thereby enabling providers to re-engage more fully in relational aspects of care [12]. In this sense, AI does not replace care but can create conditions in which human-centered care is more fully realized.

The reinterpretation of care values across phases highlights the adaptive and evolving nature of care ethics within technological environments. Attentiveness shifted toward an interpretive form that combines data-driven insights with contextual understanding. Responsibility emerged as the most vulnerable value, stabilizing only after regulatory and institutional supports matured [13]. Competence expanded beyond technical skill to encompass relational communication and interpretive expertise. Responsiveness evolved into a balanced model in which efficiency and human sensitivity coexisted. Trust, initially diminished, recovered as technological reliability improved and care structures became more transparent. Together, these transitions illustrate that care remains relational at its core, but the modalities through which relationality is enacted are transformed.

Differences between patient and provider perspectives further illuminate the relational complexity of AI-enabled care. Patients emphasized attentiveness, responsibility, and trust, reflecting their desire for stability, protection, and recognition within unfamiliar digital environments. Providers focused on competence, attentiveness, and responsibility, indicating the challenges of navigating shifting professional identities and new technological expectations. These contrasting priorities underscore that effective AI integration must address both the experiential needs of patients and the professional realities of clinicians [14].

Within the Korean healthcare context, these findings carry particular relevance. Korea’s rapid digitalization, centralized health system, and strong societal expectations for efficiency create an environment in which AI adoption accelerates quickly, but relational adaptation may lag [15]. The temporary legalization of telemedicine during COVID-19, followed by regulatory reversals, introduced additional uncertainty that shaped patient and provider experiences. This study offers insight into how Korean patients and clinicians negotiate these shifting conditions and shows that technological advancement alone does not guarantee improved care experiences; rather, ethical, relational, and institutional supports are necessary to ensure meaningful integration.

Overall, the results indicate that as AI becomes embedded within healthcare systems, the meaning of care does not disappear but evolves. Human presence, empathy, and relational understanding remain central, but they are enacted through new hybrid forms of collaboration between clinicians and technologies. These findings contribute to ongoing discussions about human-centered AI in healthcare by demonstrating that ethical and relational dimensions are not secondary concerns but essential conditions for successful technological integration [16].

### 4.1. Contextual and Temporal Influences on Experience

It is important to interpret the three-phase trajectory in light of broader contextual and temporal factors that unfolded alongside AI adoption. Regulatory changes related to telemedicine during and after the COVID-19 pandemic, shifting public health risks, and fluctuations in digital access likely shaped both patient and provider experiences independently of AI technologies themselves. For example, early disruptions in care may reflect not only technological mediation but also heightened uncertainty, infection-related anxiety, and rapidly changing institutional policies. Similarly, improvements observed in later phases may be partially attributable to regulatory stabilization, increased digital familiarity, and normalization of remote or AI-supported care practices. These findings suggest that experiences of AI-enabled care emerge through the interaction of technological innovation with regulatory, social, and temporal conditions, rather than as isolated effects of AI alone.

### 4.2. Limitations

This study has several limitations. First, the relatively small number of narratives (*n* = 25) limits statistical generalizability. However, the study aims to provide in-depth, interpretive insights into patient and provider experiences, offering analytical and conceptual transferability rather than population-level inference. Second, all narratives were drawn from Korean-language online platforms and reflect the institutional, cultural, and regulatory context of the Korean healthcare system, particularly during and after the COVID-19 pandemic. Patients’ perceptions of AI, trust in medical authority, and expectations of efficiency and accuracy may differ across healthcare systems and cultural settings. Therefore, caution is required when transferring these findings to other national or cultural contexts. Third, the data consisted of publicly available online narratives, which may privilege more vocal, digitally engaged, or emotionally expressive individuals. Experiences of patients who are less digitally literate or who engage primarily in offline clinical encounters may be underrepresented. Consequently, the discourse analyzed here reflects platform-dependent expressions of experience rather than a comprehensive account of all patient perspectives. Finally, although the study distinguished between patient and provider narratives, the inclusion of healthcare professionals’ reflections introduces an additional interpretive layer. Providers’ accounts may emphasize organizational and professional concerns that differ from patients’ lived experiences. Future studies could benefit from larger, purposefully stratified datasets that more clearly separate patient-only narratives or systematically compare stakeholder groups. In addition, the sentiment analysis relied on a standardized Korean sentiment lexicon, which may not fully capture contextual nuances, mixed emotions, or culturally embedded expressions present in patient narratives. Subtle forms of ambivalence, irony, or relational tension may therefore be underrepresented in polarity-based classifications. To mitigate this limitation, sentiment findings were interpreted in conjunction with qualitative thematic analysis rather than as standalone indicators of emotional experience. Despite these limitations, this study contributes meaningful insights into the ethical and relational dimensions of AI-enabled care by foregrounding patient-generated narratives and situating them within a care ethics framework. Given the small sample size, the findings should be understood as exploratory and interpretive rather than generalizable or statistically authoritative. The study aims to illuminate patterns of meaning and value transformation, not to provide representative prevalence estimates.

### 4.3. Implications for Design and Practice

The findings of this study offer several implications for the design and implementation of AI-enabled healthcare systems aimed at preserving human-centered care, relational engagement, and trust. First, interface design should support transparency and interpretability, enabling patients to understand how AI contributes to clinical decisions rather than experiencing it as opaque or detached from human judgment. Clear explanations, contextualized feedback, and opportunities for patient questions may help mitigate perceptions of depersonalization. Second, AI systems should be integrated into clinical workflows in ways that reduce cognitive and administrative burden for clinicians, thereby creating space for relational engagement rather than replacing it. When AI supports information synthesis or routine tasks, clinicians may have more time to attend to patients’ emotional concerns, life contexts, and values—an outcome repeatedly emphasized in patient narratives. Finally, patient communication strategies should explicitly acknowledge the collaborative role of AI and clinicians. Framing AI as a supportive tool embedded within human care relationships, rather than as an autonomous decision-maker, may strengthen trust and alleviate concerns related to responsibility, surveillance, or loss of autonomy. Together, these considerations suggest that the ethical value of AI in healthcare depends not only on technical performance but on design choices that actively sustain relational and human-centered dimensions of care [13,17].

### 4.4. Theoretical Integration with Care Ethics and Human-Centered AI

The findings of this study can be further interpreted through the lens of care ethics and human-centered AI frameworks. Core care ethics values—such as attentiveness, responsibility, competence, and responsiveness—were reflected in how patients evaluated AI-enabled care not merely in terms of efficiency or accuracy, but in relation to empathy, relational continuity, and perceived moral responsibility. Similarly, principles of human-centered AI emphasize transparency, interpretability, and alignment with human values, which resonate with patients’ concerns about trust, autonomy, and meaningful engagement identified across the three phases. Together, these theoretical perspectives help situate the empirical patterns observed in this study within broader ethical and design-oriented discourses, highlighting that the acceptability of AI in healthcare depends on its capacity to sustain relational and value-sensitive dimensions of care rather than solely optimize technical performance.

## 5. Conclusions

The findings of this study demonstrate that the integration of AI into healthcare reshapes—not replaces—the relational, ethical, and practical foundations of care. Across the three phases examined, care values initially weakened during the abrupt shift to remote interactions, shifted and diversified as AI technologies entered clinical workflows, and ultimately strengthened as digital systems stabilized and human–technology collaboration matured. These transitions reveal that attentiveness, responsibility, competence, responsiveness, and trust are not static principles but dynamic relational practices that adapt to changing technological environments.

The study contributes to the growing body of human-centered AI research by showing that patients’ experiences with AI-mediated care are grounded not only in perceptions of efficiency and accuracy but also in expectations of being recognized, protected, and understood. For clinicians, AI introduced challenges related to shifting roles and competencies, yet also enabled renewed engagement with relational aspects of care once technologies became integrated more seamlessly. Within the Korean healthcare context—characterized by rapid digitalization, high patient volumes, and evolving regulatory structures—these findings highlight the importance of aligning technological innovation with ethical clarity, relational sensitivity, and institutional support.

Ultimately, this study suggests that the future of AI-enabled healthcare depends not on technological capability alone but on the extent to which digital systems are designed and implemented to support, rather than replace, the human foundations of care. As AI becomes increasingly embedded in clinical practice, sustained attention to care ethics, patient experience, and professional identity will be essential to ensuring meaningful, trustworthy, and equitable care. Continued research, including larger-scale analyses and interdisciplinary inquiry, is needed to further understand how care values evolve across different cultural, institutional, and technological settings.

## Figures and Tables

**Table 1:** Comparison of the present study with recent key studies on patient perceptions of AI in healthcare.

Author (Year)	Country	Data Source	Methodology	Analytical Focus	Key Findings/Contribution
Smith et al. (2023) [4]	USA	Survey data	Quantitative analysis	Trust and acceptance of diagnostic AI	Patient trust varies by perceived accuracy and clinician involvement
Gundlack et al. (2025) [3]	Multiple countries	Interviews	Qualitative thematic analysis	Acceptance and challenges of AI in care	Concerns regarding transparency, autonomy, and human interaction
Hou (2025) [5]	Multiple countries	Literature review	Systematic review	Ethical concerns of AI in healthcare	Identified responsibility, trust, and transparency as key ethical issues
This study	Korea	Online narratives (2020–2025)	Text mining + care ethics framework	Longitudinal evolution of care values	Identifies a three-phase evolution of care values (disruption → redefinition → restoration) in AI-mediated healthcare within a Korean context

**Table 2:** Data sources and characteristics of the narrative dataset.

Data Source	Description	Sample Size
News comments	Public perceptions and reactions	11
Healthcare provider forums	Clinical experiences and frontline observations	8
Patient communities	Direct patient experiences and concerns	6
Total		25

**Table 3:** Characteristics of care changes across three phases.

Phase	Key Change Pattern	Dominant Value Shifts	Representative Quotations
Phase 1 (2020–2021) Disrupted Care	Weakened (66.7%)	Decline in attentiveness, reduced trust, and unclear boundaries of responsibility.	I feel like the doctor cannot fully see my condition through the screen (Patient) There is no time to hold the patient’s hand or offer comfort anymore (Nurse)
Phase 2 (2022–2023) Re-definition & Uncertainty	Mixed patterns of weakened, transformed, and emerging forms	Ambiguity in roles and responsibility; divergence between trust in technology and trust in clinicians	If AI makes a mistake, who is responsible? (Patient) My role is shifting toward interpreting AI results for patients. (Physician)
Phase 3 (2024–2025) Integration & Stabilization	Strengthened (100%)	Strengthened human–AI collaboration; restoration of relationship-centered care	AI gives accurate findings, and the doctor’s explanation makes me feel reassured. (Patient) I now have more time to understand patients’ emotions and everyday lives. (Nurse)

Note. Percentages are presented for descriptive purposes only and reflect patterns within a small qualitative dataset.

**Table 4:** Phase-based changes in care values interpreted through care ethics.

Care Value	Phase 1 (2020–2021)	Phase 2 (2022–2023)	Phase 3 (2024–2025)	Interpretation Summary
Attentiveness	Weakened—reduced attentiveness due to screen-based consultations	Emerging form—combination of AI analytic input and clinician interpretation	Strengthened—coexistence of technical accuracy and relational attentiveness	Attentiveness is reconfigured into an interpretive form that integrates human and technological insight
Responsibility	Unclear—boundaries of accountability not well defined	Unresolved—insufficient structure for assigning responsibility in AI-supported diagnosis	Strengthened—institutional and regulatory clarification of accountability	Responsibility is the value that stabilizes most slowly in AI-enabled care
Competence	Weakened—limited clinical capacity in remote environments	Transformed—new competencies in interpreting and applying AI outputs required	Strengthened—greater emphasis on relational, explanatory, and empathetic skills	Clinical competence shifts from diagnostic execution to interpretive and relational expertise
Responsiveness	Transformed—increased efficiency but reduced relational responsiveness	Transformed—faster diagnostic flow but reduced time for clinician–patient interaction	Strengthened—balanced integration of efficiency and human responsiveness	A new model emerges in which efficiency and relational responsiveness coexist
Trust	Weakened—diminished relational trust due to non-contact care	Ambiguous—divergence between trust in technology and trust in clinicians	Strengthened—improved technological reliability reinforces human trust	Trust recovers as technology and institutional structures stabilize

Note. Percentages are presented for descriptive purposes only and reflect patterns within a small qualitative dataset.

## Data Availability

The data supporting the findings of this study consist of publicly available, de-identified textual data. Due to platform terms-of-service restrictions and privacy considerations, the original narrative data cannot be shared. Aggregated results and methodological documentation are available upon request.
